# Therapeutic Drug Monitoring and Individualized Medicine of Dasatinib: Focus on Clinical Pharmacokinetics and Pharmacodynamics

**DOI:** 10.3389/fphar.2021.797881

**Published:** 2021-12-06

**Authors:** Shiyu He, Jialu Bian, Qianhang Shao, Ying Zhang, Xu Hao, Xingxian Luo, Yufei Feng, Lin Huang

**Affiliations:** ^1^ Department of Pharmacy, People’s Hospital of Peking University, Beijing, China; ^2^ Department of Pharmacy Administration and Clinical Pharmacy, School of Pharmaceutical Sciences, Peking University, Beijing, China; ^3^ School of Pharmaceutical Sciences, Tsinghua University, Beijing, China

**Keywords:** dasatinib, pharmacokinetics, exposure-response relationships, analytical method, therapeutic drug monitoring, individualized medicine

## Abstract

Dasatinib is an oral second-generation tyrosine kinase inhibitor known to be used widely in Philadelphia chromosome-positive (Ph^+^) chronic myeloid leukemia (CML) and Ph^+^ acute lymphoblastic leukemia (ALL). Notably, although a high pharmacokinetic variability in patients and an increased risk of pleural effusion are attendant, fixed dosing remains standard practice. Retrospective studies have suggested that dasatinib exposure may be associated with treatment response (efficacy/safety). Therapeutic drug monitoring (TDM) is gradually becoming a practical tool to achieve the goal of individualized medicine for patients receiving targeted drugs. With the help of TDM, these patients who maintain response while have minimum adverse events may achieve long-term survival. This review summaries current knowledge of the clinical pharmacokinetics variation, exposure-response relationships and analytical method for individualized dosing of dasatinib, in particular with respect to therapeutic drug monitoring. In addition, it highlights the emerging insights into several controversial issues in TDM of dasatinib, with the aim of presenting up-to-date evidence for clinical decision-making and insights for future studies.

## 1 Introduction

Dasatinib is an oral second-generation dual Src-Abl1 kinase inhibitor indicated for the treatment of adults and children with Philadelphia chromosome-positive (Ph^+^) chronic myeloid leukemia (CML) and Ph^+^ acute lymphoblastic leukemia (ALL) with resistance or intolerance to prior therapy including imatinib, and has become the first-line treatment for CML (Lindauer and Hochhaus, 2018). Dasatinib is 325 times as potent as imatinib in inhibiting unmutated BCR-ABL kinase *in vitro*, and has inhibitory activity against the majority of imatinib-resistant BCR-ABL mutants (Lombardo et al., 2004). Besides, dasatinib has activity in multiple other kinases, including c-KIT, PDGFRβ, and ephrin receptor kinases (Lindauer and Hochhaus, 2018).

Dasatinib has showed an association between exposure and response. As pharmacokinetic (PK) exposure of dasatinib varies highly among patients, some patients may be exposed to the risk of therapeutically relevant toxicity due to high exposure, while others may suffer from suboptimal efficacy resulted from low exposure (Verheijen et al., 2017). Additionally, dose reduction has become a target for further treatment in some patients with CML having achieved cytogenetic and hematological responses, which can not only reduce the incidence and severity of toxicities, but also lighten the financial burden on patients. However, after the responses have been achieved, we do not have a clear indicator to guide reduction for achieving maximum benefits under minimum dose. Although dasatinib has showed satisfactory efficacy, it still has problems worried us.

Therapeutic drug monitoring (TDM) is an effective tool aimed at optimizing a patient’s drug regimen based on biological fluids concentrations of the drug. Several pieces of evidence suggest potential benefits of TDM for the treatment of cancer with tyrosine kinase inhibitors (TKIs). For the treatment dilemma that dasatinib is currently facing, TDM is likely to be an effective assistant strategy to contribute to the solution of problems in clinical practice. Nevertheless, no consensus has been reached on the TDM of dasatinib, including whether it is carried out routinely, monitoring indicators, target ranges and feasibility, etc. The focus of this review is to discuss the problems above in terms of factors affecting exposure of dasatinib, exposure-response relationships for dasatinib in previous studies, as well as analytical method.

## 2 Pharmacokinetics Variability

Very high interpatient variability of dasatinib exposure was observed on maximum plasma drug concentration (C_max_), 70–80%, and on area under the plasma concentration-time curve (AUC), 40–54% (Wang et al., 2013; Ishida et al., 2016; Chandani et al., 2017). Moreover, one study suggested that the variability in exposure of dasatinib was greater within subjects than between subjects (Dai et al., 2008). Many factors that lead to the high variability of dasatinib by influencing the PK process of dasatinib need to be considered.

### 2.1 Absorption

Dasatinib is rapidly absorbed following oral administration with time to C_max_ (T_max_) ranging from 0.5 to 6 h (European Medicines Agency, 2006). With the emergence of more studies, T_max_ values observed among subjects ranged from 0.28 to 6.3 h (Horinkova et al., 2019). The oral bioavailability was rather low in preclinical studies with values ranging from 45 to 51% (Luo et al., 2006a), whereas other studies showed bioavailability from 14 to 34% (Kamath et al., 2008b). Although a high-fat meal increases the mean AUC of dasatinib by 14% after a single dose of 100 mg (Bristol-Myers Squibb, 2017), the change is not clinically significant.

The most important factor impacting dasatinib absorption is gastric pH. Dasatinib (pK_a_ = 3.1, 6.8, and 10.8) is a weak base drug and dissolves better in an acidic environment and precipitates in the small intestine (Tsume et al., 2015) (BCS/BDDCS II) (Budha et al., 2012). An extremely low AUC (54.1 ng⋅h/mL) and C_max_ (8.3 ng/ml) were reported in a patient with a history of total gastrectomy surgery, showing that the influence of gastric pH on reduced absorption of dasatinib through total gastrectomy surgery for the first time (Iwamoto et al., 2019). An *in vivo* study had shown that the elevated gastric pH range of 4.0–6.0 would significantly reduce the solubility of dasatinib (Tsume et al., 2015). Due to the pH-dependent solubility, all co-administration agents regulating gastric pH may have a great impact on the oral bioavailability of dasatinib, including proton pump inhibitors (PPIs), H_2_-receptor antagonists (H_2_RAs), antacids, pentagastrin and betaine HCl (Table 1).

**TABLE 1:** Effect of gastric acid pH modulators on the oral absorption of dasatinib.

Dasatinib (dose regimen)	Gastric acid pH modulator (dose)		Subject (N)	Change		Comment	References
				AUC	C_max_		
100 mg, QD	Day 1: dasatinib; Days 2–5: omeprazole (40 mg); Day 6: dasatinib and omeprazole (40 mg)		Healthy subjects (N = 13)	↓ 43%	↓ 42%	AUC_inf_; C_max_	Clinicaltrials.gov, (2009); Budha et al., (2012)
50 mg, Q12H	Famotidine (40 mg) 2 h after the evening dose of dasatinib		Healthy subjects (N = 21, crossover study)	No significant change		AUC_0-12_; C_max_	Eley et al. (2009)
	Famotidine (40 mg) 10 h before the morning dose of dasatinib			↓ 61%	↓ 63%		
	Maalox^a^ (30 ml) 2 h prior to dasatinib			↑ 5%^b^	↑ 26%		
	Maalox^a^ (30 ml) co-administered with dasatinib			↓ 55%	↓ 58%		
20–140 mg, Q12H	Lansoprazole (30 mg) or famotidine (20–40 mg/d) or nizatidine (300 mg/d)		CML or Ph^+^ ALL (N = 12)	Median: ↓ 58%^b^	Median: ↓ 72%^b^	AUC_0-4_ss; C_2_ss	Takahashi et al. (2012a)
70 mg, BID	Famotidine (20 mg)		Ph^+^ ALL (N = 1)	↓ 72%^b^	/	AUC_0-12_	Matsuoka et al. (2012)
3.4 ± 0.1 mg, QD (based on a 50 mg human dose)	Famotidine (10 mg/kg, iv) 2 h prior to dasatinib		Rats (N = 5)	↓ 78%	↓ 82%	AUC_inf_; C_max_	Lubach et al. (2013)
	Pentagastrin (0.25 mg/kg, ih) 2 h prior to dasatinib		Rats (N = 5)	↓ 29%	↓ 43%		
100 mg, QD	Pretreatment: Rabeprazole (20 mg, BID) for 3 days	Rabeprazole (20 mg), gastric pH ≥ 4 for at least 15 min before dasatinib	Healthy subjects (N = 12, crossover study)	↓ 78%	↓ 92%	AUC_inf_; C_max_	Yago et al. (2014)
		Betaine HCl (1,500 mg) 5 min prior to dasatinib		↑ 5%	↑ 21%		
50 mg, QD	Esomeprazole (40 mg)		Ph^+^ ALL (N = 1)	↓ 32%	↓ 56%	AUC_0-6.5_; C_max_	Pape et al. (2016)

QD, once daily; BID, twice daily; Q12H, every 12 h; N, number of subjects; iv, intravenous injection; ih, hypodermic injection; ss, steady state.

a Maalox, Aluminum hydroxide/magnesium hydroxide–containing antacid.

b Calculated from the data in the reference; /, unmentioned in the reference.

PPIs (omeprazole, lansoprazole, rabeprazole and esomeprazole) which cancer patients often take for palliation of the gastroesophageal reflux, dyspepsia, and gastritis, have the effect of decreasing dasatinib exposure, which has been demonstrated in several studies. In study of Budha et al., after 4 days administration of omeprazole (40 mg/d), dasatinib (100 mg/d) was administered concomitantly with omeprazole on the next day. The C_max_, AUC from time zero extrapolated to infinite time (AUC_inf_) and relative bioavailability (F_R_) of dasatinib in healthy subjects were reduced by 42, 43 and ∼40%, respectively (Wang et al., 2008; Budha et al., 2012). In another study, 6 healthy subjects who began with a pretreatment of 20 mg rabeprazole twice daily for 3 days, were given a morning dose on the morning of the fourth study day before 100 mg dasatinib, and significant reductions in dasatinib C_max_ and AUC_inf_ of 78 and 84% were observed (Yago et al., 2014). One case report has also indicated that after esomeprazole discontinuation for 4 days, C_max_ and the estimated AUC_0-6.5_ increased from 23.1 to 52.0 ng/ml and from 89.6 to 130.6 ng⋅h/mL, respectively (Pape et al., 2016).

H_2_RAs significantly reduce dasatinib exposure both in animals and human. In the preclinical study, when the rats were given 3.4 mg of dasatinib (equal to 50 mg in human) along with famotidine (10 mg/kg) injected 2 h prior to dasatinib dosing, the AUC_inf_ of dasatinib approximately declined 4.5-fold compared with the control group (Lubach et al., 2013). In healthy subjects, a dramatic decrease in C_max_ and AUC_0-12_ of dasatinib of 63 and 61%, respectively, was observed even though they were taking famotidine 10 h earlier than dasatinib (Eley et al., 2009). Same trend was also noticed in a patient with Ph^+^ ALL (Matsuoka et al., 2012). On the contrary, famotidine taken 2 h post dasatinib had no effect on the PK of dasatinib, suggesting that administration of famotidine 2 h following dasatinib mitigated the interaction between these two drugs (Eley et al., 2009).

Similarly, when healthy subjects received 30 ml of aluminum hydroxide/magnesium hydroxide–containing antacid (Maalox) co-administered with dasatinib, the AUC_0-12_ and C_max_ of dasatinib were decreased drastically by 55 and 58%, respectively. Whereas the antacid Maalox, taken 2 h before dasatinib, showed no significant change (Eley et al., 2009).

Interestingly, pentagastrin, which is well-known to stimulate acid secretion in mammals, was found to reduce exposure of dasatinib in rats. A reasonable explanation may be that dasatinib is probably fully dissolved in the stomach because of the high solubility at low pH (below ∼4), indicating that as the drug passes through the intestinal tract (pH increases to 5–7), systemic absorption of dasatinib decreases owing to rapid supersaturation in the gut and possible precipitation. In this study, the gastric juice of fasted rats was highly acidic, with a pH around 2, which is close to the value of the pentagastrin pretreatment group (Lubach et al., 2013). That means patients had better not take dasatinib with a prolonged fast, although food has no effect on the absorption of dasatinib.

In order to mitigate the interaction between PPIs and dasatinib, two studies reported the effects of betaine HCl and acidic beverages. Patients pretreated with rabeprazole received betaine HCl (1,500 mg) 5 min pre-dasatinib and it was able to restore dasatinib AUC_inf_ and C_max_ to 1.05, and 1.21-fold of the control treatment with dasatinib alone. That means that the reduced exposure to dasatinib induced by rabeprazole can be reversed through coadministration with betaine HCl (Yago et al., 2014). Furthermore, it was found that the effect of concomitant strong acid-reducing agents on ketoconazole, posaconazole, and erlotinib absorption may be offset by coadministration of acidic beverages such as glutamic acid, dilute hydrochloric acid, or carbonated beverages (e.g., cola). Since both dasatinib and these drugs require an acidic environment for optimal absorption, thus, Knoebel et al. suggest that coadministration of acidic beverages such as cola with dasatinib may be a rational option for patients who require potent acid-reducing agents, especially for PPIs (Knoebel and Larson, 2018).

### 2.2 Distribution

Approximately 96% of dasatinib (93% of its active metabolite) is bound to human plasma proteins (mainly to albumin) *in vitro* (Bristol-Myers Squibb, 2017). And the apparent volume of distribution is 2505 L (Bristol-Myers Squibb, 2017), demonstrating that dasatinib is well distributed into tissues. As we learned, drugs with moderate to high affinity for the same binding sites can have an effect on free drug serum concentrations by competing protein binding sites. For instance, aspirin, displaced valproate from protein binding sites and the free fraction of valproate increased significantly (Orr et al., 1982). Regrettably, no study on the protein binding of dasatinib was found. In addition, there was a negative correlation between the amount of albumin and valproate concentration (Lai et al., 2020). Additional attention should be paid to patients with low albumin levels due to complicated cirrhosis or hypoproteinemia on account of the high binding of dasatinib to plasma proteins.

The cellular uptake of dasatinib is not dependent on drug transporters. The intracellular uptake and retention (IUR) of dasatinib was linear over the range of drug concentrations tested at 4 and 37°C temperatures, suggesting that the cellular uptake is mainly a passive process (Giannoudis et al., 2008; Hiwase et al., 2008). Accordingly, dasatinib uptake is not dependent on hOCT1 (hOCT2 and hOCT3, too (Kamath et al., 2008a)) in contrast to imatinib, even if dasatinib is a substrate for hOCT1 (Giannoudis et al., 2008).

The cellular efflux of dasatinib is partially regulated by drug transporters, which has been confirmed by studies both *in vivo* and *in vitro*. The effects of several efflux proteins belonging to the ATP-binding cassette transporter family on dasatinib diffusion have been reported in multiple studies, including ABCB1 (MDR1/P-gp), ABCG2 (BCRP) and ABCC6 (MRP6). It had been shown that dasatinib was a substrate for both P-gp and BCRP by cell models (Giannoudis et al., 2008; Hiwase et al., 2008) as well as directly measuring intracellular dasatinib levels (Chen et al., 2009; Hegedus et al., 2009). Overexpression of ABCB1 or ABCG2 protein reduced dasatinib IUR, resulting in an increase in the IC_50_ of dasatinib, which could be modulated with inhibitors (Hiwase et al., 2008; Chen et al., 2009). Likewise, a study conducted in BCR-ABL1^+^ cell lines indicated that inhibition of ABCC6 reduced dasatinib efflux, leading to a significant decrease in IC_50_ of dasatinib (Eadie et al., 2018).

The central nervous system (CNS) delivery of dasatinib is predominantly subject to the limitations of P-gp and BCRP. *In vivo*, there was no difference in brain accumulation of dasatinib between *Abcg2* ^*−/−*^ mice and wild-type (WT) mice, but it was 3.6-fold and 13.2-fold increase in *Abcb1a/1b* ^*−/−*^ and *Abcb1a/1b;Abcg2* ^*−/−*^ mice, respectively (Lagas et al., 2009), which was confirmed in study by Chen et al. (2009) These results show that P-gp likely exerts a leading role in limiting CNS delivery of dasatinib, whereas BCRP alone shows no such effect on CNS transport of dasatinib. Notably, similar results were reported for imatinib and lapatinib. It suggests that there appears to be a “synergistic” activity of P-gp and BCRP, with the highest efflux activity exhibited by the combination of these two transporters (Bihorel et al., 2007; Chen et al., 2009; Lagas et al., 2009; Polli et al., 2009). The CNS concentration in patients was rarely examined and mostly was undetectable, even if the samples were collected in the absorptive phase (Porkka et al., 2008). In 7 patients with CML or Ph^+^ ALL (including adult and pediatric), the brain-to-plasma concentration (B/P) ratio was detected (0.01, 0.03, 0.03, 0.04, 0.05, 0.08, and 0.28) (Porkka et al., 2008; Zhou et al., 2013). And the B/P ratios of AUC_0–24_ in 2 pediatric patients with diffuse intrinsic pontine glioma were 0.028 and 0.016 (Broniscer et al., 2013). The finding that P-gp and BCRP have a combined effect on CNS delivery of drug may have significant implications for the treatment of CNS leukemia.

PPIs are substrates and inhibitors of P-gp (Pauli-Magnus et al., 2001). High concentrations of pantoprazole and esomeprazole (1 and 2 mM) resulted in a significant increase of dasatinib IUR in ABCB1 overexpressing cells (Hiwase et al., 2010), which was probably due to the fact that PPIs inhibited the function of P-gp to pump the dasatinib out of the target cells. However, this concentrations at clinically relevant doses is much lower than that of 1 and 2 mM and thus does not achieve the effect of P-gp inhibition. There are other P-gp inhibitors which also have no *in vitro* and *in vivo* data, including cyclosporine, itraconazole, calcium antagonists, antiarrhythmic drugs and macrolides antibiotics. More care still should be taken if co-administered with these drugs.

### 2.3 Metabolism

Dasatinib is metabolized in humans, primarily by CYP3A4, and flavin-containing monooxygenase 3 (FMO-3) and uridine diphosphate-glucuronosyltransferase (UGT) enzymes are also involved in the formation of dasatinib metabolites (Bristol-Myers Squibb, 2017). Routes of metabolism include hydroxylation, N-dealkylation, N-oxidation, alcohol oxidation and direct glucuronide or sulphate conjugation (Horinkova et al., 2019). There are five phase I circulating metabolites: M4, M5, M6, M20 and M24, among which M20 and M24 represent 45 and 25% of the AUC_0-24_ of dasatinib, respectively. The primary active metabolite, M4 (N-dealkylated) whose antiproliferative activity *in vitro* is similar to that of dasatinib represents only 5% of dasatinib AUC(Leveque et al., 2020).

The coadministration of CYP3A4 inhibitors or inducers lead to varying degrees of fluctuation in dasatinib plasma concentrations. The mean C_max_ and AUC of dasatinib increased by 4-fold and 5-fold, respectively, when used in combination with ketoconazole (strong CYP3A4 inhibitor) (Bristol-Myers Squibb, 2017). Similarly, other moderate or strong CYP3A4 inhibitors used frequently in patients with CML may have the same effect, such as, aprepitan, grapefruit juice, macrolides antibiotics (clarithromycin and erythromycin), azole antifungal agents (itraconazole, voriconazole, and posaconazole) and so on. In opposite, the coadministration of rifampin (strong CYP3A4 inducer) decreased the mean C_max_ and AUC of dasatinib by 81 and 82%, respectively (Bristol-Myers Squibb, 2017). Other CYP3A4 inducers include antiepileptic drugs, dexamethasone and herbal preparations such as St John’s wort (known as *hypericum perforatum* in China) and ginseng (Leveque et al., 2020).

The data from European public assessment reports (EPARs) show a high degree of absorption of dasatinib with the fraction of the dose absorbed (f_abs_) of at least 70%. However, due to the low absolute bioavailability (34%), a considerable amount of first-pass metabolism can be predictably expected. So if CYP3A4 has a 50% or greater contribution to the overall clearance of dasatinib, then a phenotype analysis might be essential for dose adjustment to enable individualized medicine (Mikus and Isabelle Foerster, 2017).

### 2.4 Excretion

Elimination is primarily via the feces. Besides, the bile also plays a role. Following a single radiolabeled dose of oral dasatinib, 4% of the administered radioactivity was recovered in the urine and 85% in the feces within 10 days, and unchanged dasatinib was in the minority. The mean terminal half-life of dasatinib is 3–5 h (Bristol-Myers Squibb, 2017).

The gut microbiome has been recognized as the second human genome. So, it’s not surprising that large interpatient variability of the gut ecosystem has been found. Additional data has become available showing that except liver, the gut microbiome can also directly influence an individual’s response to a specific drug by enzymatically transforming the drug’s structure and altering its bioavailability, bioactivity or toxicity (Doestzada et al., 2018; Weersma et al., 2020). As the majority of dasatinib is excreted out of body through feces, it is worthy of further investigation for a complex interaction of dasatinib with the gut microbiome.

### 2.5 Pharmacogenetics

The pharmacogenetics of dasatinib are rarely reported. Although to date we have not found any evidence of a relationship between *CYP3A4* gene polymorphisms and dasatinib PK, the effects of *CYP3A4* gene polymorphisms and its regulation and expression on PK of drugs have been extensively investigated. The CYP3A4*1G allele with high frequency in Asians was suggested to decrease CYP3A activity and fentanyl consumption (Wei et al., 2010). Additionally, in clinic observation of kidney transplantation, CYP3A4*22 carriers required less Tacrolimus dose to achieve the target exposure compared with CYP3A4*1/*1 carriers (Yu et al., 2018).

The 3 most relevant *ABCB1* gene polymorphisms are: 1236C > T, 2677G > T/A, and 3435C > T. One study reported *ABCB1* TTT haplotype (1236T, 2677T, 3435T) led to significantly lower intracellular accumulation of dasatinib (Skoglund et al., 2013). Moreover, an *in vitro* study reported that the *ABCB1* 1199A variant was associated to a higher ABCB1 efflux activity, particularly toward imatinib and dasatinib (Dessilly et al., 2016).

Taken together, all these results suggest that polymorphisms of metabolic enzymes and transporters may have a potential impact on dasatinib exposure in plasma and further studies with large sample size are needed to confirm this.

### 2.6 Special Populations

Age (15–86 years old), sex, and renal impairment (creatinine clearance 21.6 ml/min to 342.3 ml/min as estimated by Cockcroft Gault) have no clinically relevant effect on the PK of dasatinib, according to the prescribing information (Bristol-Myers Squibb, 2017).

#### 2.6.1 Pediatric Patients

The PK of pediatric is very similar to that of adults. In pediatric patients with a dosing regimen of 60 mg/m^2^, the model simulated geometric mean (coefficient of variation, CV%) steady-state plasma average concentrations of dasatinib were 14.7 (64.6%) ng/mL (for 2 to <6 years old), 16.3 (97.5%) ng/mL (for 6 to <12 years old), and 18.2 (67.7%) ng/mL (for 12 years and older). Because of the difficulty in swallowing tablets, some prefer dispersed tablets, which have an estimated 36% lower bioavailability than intact tablets (Bristol-Myers Squibb, 2017).

#### 2.6.2 Patients With Hepatic Impairment

Compared to subjects with normal liver function, patients with moderate hepatic impairment (Child-Pugh B) had decreases in mean C_max_ by 47% and mean AUC by 8%. Patients with severe hepatic impairment (Child-Pugh C) had decreases in mean C_max_ by 43% and in mean AUC by 28% compared to the subjects with normal liver function (Bristol-Myers Squibb, 2017). No dose adjustment for patients with hepatic impairment is currently recommended.

## 3 Exposure-Response Relationships

### 3.1 Exposure-Efficacy

In preclinical studies (both *in vivo* and *in vitro*), it was suggested that the efficacy of kinase inhibition correlated with dasatinib exposure. In BCR-ABL-positive cell lines exposed to gradient concentrations of dasatinib (from 0.5 to 150 nM), phospho-BCR-ABL/phospho-CrkL (p-CrkL) levels were considerably diminished and apoptosis levels were increased with elevated concentrations of dasatinib (Copland et al., 2006; Shah et al., 2008b; Snead et al., 2009; O’hare et al., 2013), which was also shown in SRC-expressing cells (Luo et al., 2008). Results of validation in primary CML cells from CML patients were similar to those of cell lines (Shah et al., 2008b; Luo et al., 2008; Snead et al., 2009). In a phase I study using peripheral blood mononuclear cells (PBMC) obtained from 5 dasatinib-treated CML patients, p-CrKL decreased in a dose-dependent manner after 4 h of the initial dose but was largely recovered after 8 h, coinciding with the decline in dasatinib serum levels measured in these same patients (Talpaz et al., 2006).

Besides, in tumors from mice bearing human tumor xenografts, tumoral phospho-BCR-ABL/phospho-SRC was dose-dependently inhibited (a single oral dose ranging from 1.25 to 50 mg/kg), and directly related to the plasma concentrations of dasatinib (Luo et al., 2006b; Luo et al., 2008). Notably, two studies reported that practically undetectable p-CrkL levels (O’hare et al., 2013) or near-maximal apoptosis levels (Snead et al., 2009) were observed when exposed to 100 nM (∼48.8 ng/ml) dasatinib. What’s more, Shah et al. found that 100 nM dasatinib exposure killed about 90% of the cells as short as 20 min, even for imatinib-resistant (except for T315I) (Shah et al., 2008b). These results suggest that transient potent inhibition (>50 ng/ml) is sufficient to commit CML cells to apoptosis.

To the best of our knowledge, few clinical studies have shown that there is some certain link between exposure and efficacy. Plasma concentration at 2 h (C_2_), C_max_ and AUC_0-4_ detected in patients with T315I were significantly lower than those without. The median C_max_ in patients with T315I and without were 43.8 ng/ml and 112.4 ng/ml, respectively (*p = 0.0242*) (Takahashi et al., 2012b). As a consequence, a low exposure to dasatinib may be associated with the emergence of BCR-ABL mutations, including T315I. In a prospective cohort study of 10 CML patients receiving dasatinib 100 mg once daily, analysis by Iwamoto et al. revealed that the cut-off value of dasatinib AUC and C_max_ for achieving major molecular response (MMR) within 6 months were 336.1 ng·h/ml and 69.2 ng/ml, respectively, and the accuracy ratio to predict MMR was 88.9% for the AUC, and 77.8% for the C_max_ (Iwamoto et al., 2019). It is worth noting that the monitoring of AUC cannot be generalized in clinical practice because of its operational complexity, although it is the best PK parameter to characterize dasatinib exposure. Compared with AUC, C_max_ is a more applicable parameter for prediction of efficacy.

The study by Wang et al. using data from 567 Ph^+^ CML subjects indicated that achieving major cytogenetic response (MCyR) was most closely related to increasing wC_avg_ss, so from the perspective of this study, the most significant predictor of MCyR was wC_avg_ss (Wang et al., 2013). Despite wC_avg_ss suggested to predict efficacy, TDM based on wC_avg_ss is not feasible clinically (Yu et al., 2014).

In aggregate, it is important that transient potent dasatinib concentration of 100 nM achieves inhibition of BCR-ABL and the effect can last for several hours, that’s why dasatinib is taken once daily in spite of the short half-life. In addition, although continuous low-level exposure can achieve a similar efficacy of BCR-ABL inhibition, a relatively high exposure is needed for reducing the risk of developing BCR-ABL point mutations. Vainstein et al. found higher inhibitory potential at peak concentration (IPP), which integrated IC_50_, slope, and C_max_, correlated with improved complete cytogenetic response (CCyR) rates in CML patients treated with dasatinib (Vainstein et al., 2013), which confirmed the importance of C_max_ laterally. A study has proved that the C_2_ concentration, not concentration at 1 h (C_1_) or 4 h (C_4_), had a higher correlation with the measured AUC_0–4_ of dasatinib using 34 PK profiles (*r = 0.9419, p < 0.0001*) (Takahashi et al., 2012a). The monitoring of C_2_ concentration is easily achieved clinically to predict whether an enough C_max_ will be obtained.

On the whole, the monitoring of dasatinib C_max_/C_2_ level does make sense. It is generally accepted that a relatively high C_max_ level should be maintained to ensure the clinical efficacy and to reduce the risk of dasatinib resistance. Based on current limited evidence, it is recommended to maintain C_2_ concentration at least ≥50 ng/ml.

Additionally, dasatinib showed the special capacity to induce immunomodulation. The present *in vitro* studies indicated that dasatinib dose-dependently inhibited the proliferation and function of CD4^+^CD25^+^ regulatory T cells (Tregs) (Fei et al., 2009), meanwhile it enhanced the expansion of large granular lymphocytes [LGLs, mono- or oligoclonal CD8^+^T cells, γδT cells, and natural killer (NK) cells] (Uchiyama et al., 2013). This effect was also confirmed using a collection of 37 leukemia patients that the blood counts closely mirrored dasatinib plasma concentrations (Mustjoki et al., 2013). Importantly, most of the dasatinib discontinuation trials showed that increased LGLs levels and reduced immune suppressive Treg levels in dasatinib treatment interrupted patients were linked to better prognosis and treatment-free remission (TFR) successes (Hughes and Yong, 2017; Climent and Plana, 2019). Therefore, we reasonably speculate that the exposure of dasatinib is closely related to TFR. However, no studies currently have been conducted to determine whether dasatinib exposure levels correlate with successful TFR. But it is highly worthy of investigation and discussion, and we expect that such research will emerge in the future.

### 3.2 Exposure-Safety

Reviewing data from several clinical studies, it was found that pleural effusion (PE), an adverse event, was intimately associated with dasatinib treatment, and was the primary reason for discontinuation. In DASISION study, fluid retention (all grades) occurred more frequently with imatinib than with dasatinib (42 vs. 19%), yet it’s a remarkable fact that PE was reported only in the dasatinib group: 26 patients (10%) (Kantarjian et al., 2010). Subsequently, it was also identified that the incidence of PE was related to the dasatinib dose regimens, with the lowest of the 100 mg once daily regimen (Shah et al., 2008a; Shah et al., 2016) (Table 2).

**TABLE 2:** Overview of outcomes for patients administrated different oral dose of dasatinib.

Study	Dose	Patient (N)	Efficacy, %					Safety, %
			OHR	CHR	MaHR	CCyR	MCyR	Pleural effusion (all grades)
Cortes et al. (2007)	70 mg, BID	CML-BC (N = 116)	47	26	33	33	38	23
Guilhot et al. (2007)	70 mg, BID	CML-AP (N = 107)	81	39	64	24	33	23
Hochhaus et al. (2007)	70 mg, BID	CML-CP (N = 186)	/	90	/	/	52	19
Kantarjian et al. (2007)	70 mg, BID	CML-CP (N = 101)	/	93	/	40	52	17
Shah et al. (2010)	100 mg, QD	CML-CP (N = 167)	/	92	37	50	63	14
	50 mg, BID	CML-CP (N = 168)	/	92	38	50	61	23
	140 mg, QD	CML-CP (N = 167)	/	87	38	50	63	25
	70 mg, BID	CML-CP (N = 168)	/	88	38	54	61	23

/, unmentioned in the reference.

OHR, overall hematologic response; CHR, complete hematologic response; MaHR, major hematologic response; CCyR, complete cytogenetic response; MCyR, major cytogenetic response; QD, once daily; BID, twice daily; BC, blast crisis; CP, chronic-phase; AP, accelerated-phase.

A phase II study (OPTIM) reported that after CML patients with a trough plasma concentration (C_0_) value ≥3 nmol/L (about 1.5 ng/ml) were randomly assigned between the continuation of dasatinib 100 mg/d (control arm: *n* = 42) and a dose reduction strategy (TDM arm: *n* = 38), the TDM arm had a significantly lower cumulative incidence of PE (12 vs. 39%) and discontinuation rates (21 vs. 36%) compared to the control arm by 36 months. It was encouraging that molecular responses evaluated during 3 years were found to be similar in both arms (Rousselot et al., 2021). In study by Mizuta et al. (2018), CML patients administered dasatinib once daily at a dose of 100 mg (*n* = 27) or 50 mg (*n* = 5) had a median C_0_ of dasatinib of 1.4 ng/ml, with no significance in PE rate between high C_0_ group (≥1.4 ng/ml) and low C_0_ group (<1.4 ng/ml) (31 vs. 19%). Nonetheless, after adjusting for dasatinib dose (g) and body weight (kg) (C_0_/D/W), higher median C_0_ was correlated with the incidence of the dasatinib interruption/reduction in treatment. These results suggest that dose optimization by C_0_ assessment using TDM, a valuable “PE prediction tool” (Rousselot et al., 2021), reduces the risk of exposure to PE while ensuring the efficacy.

Wang et al. (2013) also found that patients with 100 mg once daily schedule had the lowest PE rate (11%) and steady-state C_0_ of dasatinib (2.61 ng/ml). And the C_0_ was identified as the most significant predictor of PE in the Cox proportional hazards model (hazard increased 1.22-fold for every 1 ng/ml increase in C_0_). Based on Wang et al.’s study (Wang et al., 2008), Yu et al. (2014) defined a dose interruption rate of about 50% as a non-acceptable cut-off, then the C_0_ should not exceed 2.5 ng/ml in chronic phase CML patients. However, this cut-off value should be interpreted with caution in the clinic, as it is derived from a mixed dosing regimen (*n* = 567). What’s more, the analysis by Verheijen et al. (2017) indicated that across all kinase inhibitors, the target exposure fitted 81.7% of the population exposure and supported the argument that in the absence of a definitive TDM target, the geometric mean C_0_ of dasatinib [2.61 ng/ml for 100 mg once daily, *n* = 146 (Wang et al., 2013)], representing the CML population average, could be an alternative. There was an additional viewpoint from Mirua et al. who suggested the C_0_ cut-off value of 4.33 ng/ml (median) from the regression model studied by Wang et al. (2013) be determined as the minimum toxic concentration (MTC) to avoid PE (Miura and Takahashi, 2019). On balance, the above recommendations on the target range for dasatinib C_0_ are derived from single data source (all based on Wang et al.’ study), and as such need to be supported by additional evidence.

Age was a major risk factor for PE, which was confirmed in several studies (Wang et al., 2013; Mizuta et al., 2018; Rousselot et al., 2021). And it was found that this effect was driven by PK parameters (Wang et al., 2013). Patients with high median age had a higher level of C_0_, and so did a higher incidence of PE. As a result, the therapeutic window of elderly patients may be narrower relative to younger patients, and these patients may need more intensive monitoring.

All things considered, the relationship between C_0_ of dasatinib and the occurrence of PE in patients has been basically established, i. e., maintaining a relatively low level of C_0_ can reduce the risk of PE. Meanwhile, the threshold of dasatinib C_0_ from some experts’ suggestions is concentrated at 2–5 ng/ml, despite lack of hard evidence to support it. Consequently, it is necessary to monitor C_0_ of dasatinib, but the target range needs to be further explored and confirmed.

## 4 Analytical Method

Because of the low steady-state blood concentration of dasatinib, the currently preferred analytical method for measuring concentrations of dasatinib is liquid chromatography-tandem mass spectrometry (LC-MS/MS), which has a high sensitivity for quantifying unchanged dasatinib in biological fluids. Table 3 shows studies on the LC-MS/MS analytical method of dasatinib published in recent 5 years (Table 3). Recently, some studies have reported other methods used for dasatinib, such as sequential spectrophotometric-based univariate methods (Abdelhameed et al., 2021). Most studies determined dasatinib levels with a lower limit of quantification (LLOQ) of 1 ng/ml (Couchman et al., 2012; Furlong et al., 2012; Birch et al., 2013), while the method of Bouchet et al. (2011) determined dasatinib levels with a lower LLOQ of 0.1 ng/mL. A study found that about 4.7% of the available dasatinib concentration measurements (contained 4044 measurements) were below the LLOQ of 1.0 ng/ml (Wang et al., 2013; Miura and Takahashi, 2016), suggesting that lower LLOQ of less than 1.0 ng/ml was a necessity.

**TABLE 3:** Overview of LC–MS/MS analytical methods of dasatinib in human plasma in recent 5 years.

References	Analtye	Analytical column	Internal standard	Calibration range, ng/mL	LLOQ, ng/mL	Extraction
Huynh et al. (2017)	Dasatinib, other 13 TKIs	Acquity UPLC BEH C18 column (2.1 mm × 50 mm; 1.9 µm)	^2^H_8_-dasatinib	1–500	0.75	PPT
Wojnicz et al. (2017)	Dasatinib, imatinib, nilotinib	Poroshell 120 EC-C18 column (2.1 mm × 75 mm, 2.7 µm)	D_8_-dasatinib	0.75–400	0.75	SPE
Zeng et al. (2017)	Dasatinib, imatinib, nilotinib	Xtimate Phenyl column (2.1 mm × 150 mm, 3 µm)	/	2–490	2	LLE
Maher et al. (2018)	Dasatinib	Acquity UPLC BEH C18 column (1.0 mm × 100 mm, 1.7 µm)	Erlotinib	1–500	1	SPE
Merienne et al. (2018)	Dasatinib, other 16 TKIs and 2 metabolites	CORTECS UPLC C18 column (2.1 × 50 mm, 1.6 µm)	^13^C_6_-dasatinib	0.1–200	0.1	SPE
Ezzeldin et al. (2020)	Dasatinib, other 6 TKIs	Acquity UPLC BEH C18 column (2.1 mm × 100 mm, 1.7 µm)	Quizartinib	5–1000	5	PPT
Koller et al. (2020)	Dasatinib, other 10 TKIs	Poroshell 120 EC-C18 column (2.1 mm × 75 mm, 2.7 µm)	D_8_-dasatinib	0.38–400	0.38	PPT; SPE
Mukai et al. (2020)	Dasatinib, other 4 TKIs and 3 active metabolites	L-column3 C18 (2.1 mm × 50 mm, 3 µm)	D_8_-dasatinib	0.5–150	0.5	SLE
Hirasawa et al. (2021)	Dasatinib, other 4 TKIs	Triart C18 MetalFree column (2.1 mm × 50 mm, 3 µm)	^2^H_8_-dasatinib	0.1–200	0.1	PPT
Llopis et al. (2021)	Dasatinib, other 8 TKIs, 2 active metabolites and 2 AAs	Acquity UPLC T3 HSS C18 column (2.1 × 100 mm, 1.8 µm)	^2^H_8_-dasatinib	1–500	1	PPT
Sumimoto et al. (2021)	Dasatinib, other 4 TKIs and 2 active metabolites	Acquity BEH C18 column (2.1 mm × 50 mm, 1.7 µm)	D_8_-dasatinib	0.2–200	0.2	SPE
Verougstraete et al. (2021)	Dasatinib, other 7 TKIs	Acquity UPLC BEH C18 column (2.1 mm× 100 mm, 1.7 µm)	D_8_-dasatinib	0.5–450	0.5	PPT

/, unmentioned in the reference.

PPT, protein precipitation; SPE, solid-phase extraction; LLE, liquid-liquid extraction; SLE, supported liquid extraction method using an ISOLUTE SLE+ column; LC–MS/MS, liquid chromatography/electrospray ionization–tandem mass spectrometry; AAs, antiandrogen drugs.

Currently, the monitoring of dasatinib uses plasma samples, which contain both free and bound fractions. However, only free drug in equilibrium with cells can exert pharmacological effects. Simultaneously, pharmacologically active free fraction may undergo significant changes due to variations in the concentration, conformation, and/or other physicochemical properties of plasma proteins (Haouala et al., 2013). Hence, free dasatinib concentration should be in consideration because of its high protein binding rate (Widmer et al., 2014). There was a study measured free and total imatinib concentrations and predicted imatinib free concentrations by an established model based on total concentrations and plasma proteins measurements (Haouala et al., 2013).

Plasma samples are collected in tubes using heparin or EDTA as anticoagulant by immediate centrifugation. Different sample preparation methods, such as protein precipitation (PPT) (Haouala et al., 2009; Huynh et al., 2017), solid-phase extraction (SPE) (Bouchet et al., 2011; Furlong et al., 2012; Wojnicz et al., 2017; Koller et al., 2020) and liquid-liquid extraction (LLE) (Couchman et al., 2012; Birch et al., 2013), have been used for TKIs. PPT, however, is at most risk of causing ion suppression in electrospray ionization (ESI), since it does not remove all endogenous compounds that interfere with ESI-LC-MS/MS analysis (Koller et al., 2020). In particular, samples will be diluted by the addition of protein precipitator, resulting in a lower concentration of the drug to be measured. Therefore, it is not suitable for samples with low concentration like C_0_ of dasatinib. LLE is characterized by clean extraction, of which multiple extraction steps are required to improve the recovery of analytes. SPE is less time-consuming and requires less solvent volume (Koller et al., 2020), which is a more costly method compared with LLE (Zeng et al., 2017). Moreover, Mukai et al. employed a supported liquid extraction method using an ISOLUTE SLE+ column which was more convenient compared to SPE (Mukai et al., 2020). Another proper alternative to SPE is thin-film solid-phase microextraction (TF-SPME) whose distinctive characteristics are high sensitivity, large extraction capacity, and minimum requisite sample pre-treatment (Khodayari et al., 2021).

## 5 Conclusion and Prospect

The findings presented in this review demonstrate that TDM of dasatinib is essential and feasible, and the clinical benefits of dasatinib TDM in individualized medicine have also been initially shown. Based on the evidence currently available, scholars suggest that maintain a relatively high level of C_2_ or C_max_ to obtain sufficient efficacy and reduce the risk of BCR-ABL mutations, and a relatively low level of C_0_ to reduce the risk of exposure to PE. Moreover, age is a factor with more attention and elderly patients may need regular monitoring compared with young patients.

At present, there is a real need to identify the range of the monitoring target and high-quality controlled studies need to be performed to confirm its appropriateness, especially for C_0_. Accordingly, with the progressing of the research, a classification refinement of dasatinib TDM is likely needed, such as diseases, ages, races and so on. Following the launch of targeted drugs with safety and efficacy, more and more patients are achieving responses, accompanied by increasing demands for dose reduction. Extending the existing knowledge with additional studies in the field of individualized medicine will open up the era of guiding reduction by TDM and implementing it into clinical practice in the foreseeable future to improve patients’ quality of life. Additionally, to facilitate clinical application, there is an inevitable need to advance technologies for feasible and accurate monitoring of drug concentrations. For example, the determination of free concentration or the free concentration modelled predicted can more accurately reflect the exposure of pharmacologically active free fraction. Overall, the strategy of TDM will help overcome difficulties of dasatinib in treatment and bring further survival benefit and a better quality of life for patients in the future.
